# Telomere maintenance mechanism subtype reveals different immune activity in vestibular schwannoma

**DOI:** 10.1007/s11060-023-04458-5

**Published:** 2023-10-21

**Authors:** Ji-Yong Sung, Jung Woo Lee

**Affiliations:** 1 Center for Genome Engineering, Institute for Basic Science, 55, Expo-ro, Yuseong-gu, Daejeon, 34126 Republic of Korea; 2 Department of Orthopaedic Surgery, Yonsei University Wonju College of Medicine, Wonju, Republic of Korea; 3 Yonsei Institute of Sports Science and Exercise Medicine, Wonju, Republic of Korea

**Keywords:** Vestibular schwannoma, Antigen presenting cells, Alternative lengthening of telomere, Telomere maintenance mechanism

## Abstract

**Background:**

The immortality of cancer cells relies on maintaining the length of telomeres, which prevents cellular senescence and enables unlimited replication. However, little is currently known about telomerase activity and the alternative lengthening of telomeres (ALT) in vestibular schwannomas. In this study we aimed to elucidate the role that telomerase and ALTs play in vestibular schwannomas.

**Methods:**

To address this gap, we conducted a study where we used the gene set variation analysis algorithm with bulk RNA-seq and single-cell RNA-seq to identify the characteristics of each group of patients with vestibular schwannomas, based on their telomere maintenance mechanism subtype.

**Results:**

Our findings suggest that patients with relatively high ALT-like groups have a better prognosis than those with relatively high telomerase groups. Specifically, we found that the high telomerase group had relatively higher antigen-presenting cell (APC) activity than the high ALT like group. At the single-cell level, microglia, neutrophils, and fibroblasts showed high telomerase activity and relatively high APC activity compared to other cell types. In addition, Schwann cells in the group with low ALT levels exhibited elevated immune activity at the single-cell level.

**Conclusion:**

These results suggest that personalized drug therapy could be developed from the perspective of precision medicine for patients with relatively high telomerase activity and a high ALT-like group.

## Introduction

Cancer cells can replicate indefinitely when a telomere maintenance mechanism (TMM) such as telomerase or the alternative lengthening of telomeres (ALT) pathway is activated [1]. Previous studies have investigated the length of telomeres and suggested the existence of a third TMM in pan-cancer research [2, 3]. At the single-cell level, we investigated telomere maintenance in various cell types [4]. Patients with sarcoma and some cancers have high ALT-risk mortality and thus require exact prognostic stratification and treatment based on ALT [5, 6]. Glioblastomas exhibit different immune signatures based on the manner in which telomeres are maintained, with macrophage tumors showing high levels of interferon-induced proteins with tetratricopeptide repeats (IFIT1-3) and telomerase-positive tumors containing macrophages upregulating MARCO, CXCL12, and sushi-repeat-containing protein X-linked 2 (SRPX2) [7]. In our previous pan-cancer study, glioblastoma multiforme (GBM) and liver hepatocellular carcinoma (LIHC) with high ALT levels had improved prognosis, whereas GBM with low ALT levels exhibited highly regulated expression of antigen-presenting cell-related genes [2]. Recently, studies in the field of neuro-oncology have shown that telomerase-negative glioblastoma cells with cancer-associated SMARCAL1 loss-of-function mutations extend telomeres and tumorigenesis [8]. However, the telomere maintenance mechanism in vestibular schwannomas, a benign neuro-oncological tumor, has not been studied. We aimed to explore TMM subtypes and their tumor immune environment for the first time in this study.

## Results

### Identification of TMM subtypes

**Fig. 1:**
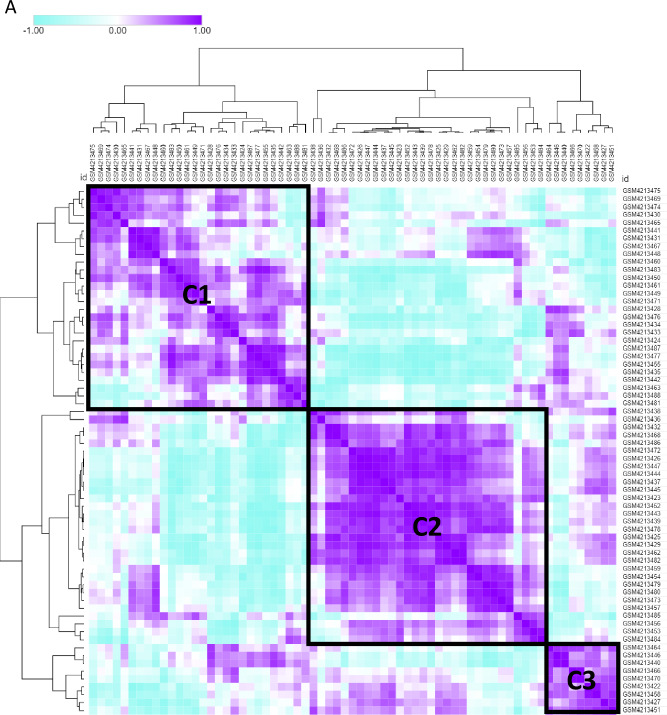

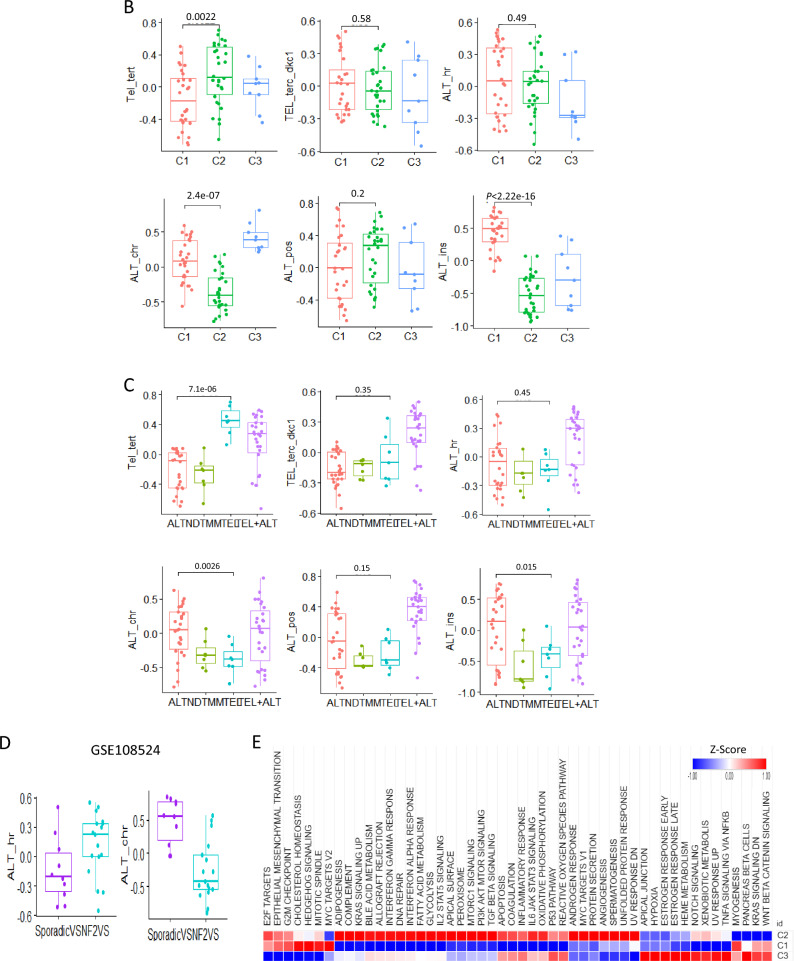

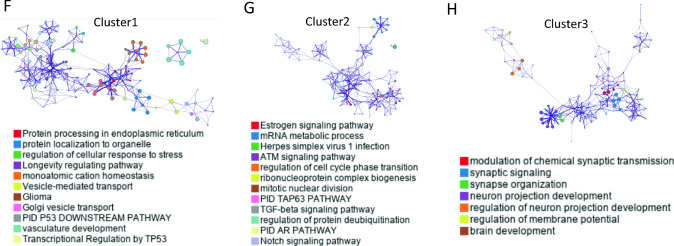
Identification of telomere maintenance mechanism (TMM) subtypes **A** Heat map of hierarchical clustering for telomere maintenance mechanism activity **B** Box plot of telomere maintenance mechanism activity in three clusters **C** Box plot of telomere maintenance mechanism activity in four TMM types (ALT: alternative lengthening of telomere, NDTMM: non-defined telomere maintenance mechanism, TEL: telomerase, TEL + ALT: telomerase and alternative lengthening of telomere) **D** Box plot for ALT activity between sporadic VS and NF2 VS in GSE108524 **E** Heatmap of cancer hallmark for three clusters. (**F**~**H**) Network for enriched biological pathway for each cluster

We used bulk RNA-seq data from patients with vestibular schwannomas to stratify patients based on their telomere maintenance pathway activity. The TMM gene set used in previous studies [2, 3] was employed to distinguish the TMM subtypes. We constructed a correlation matrix based on the unique distribution of gene signature enrichment across patient samples. Hierarchical clustering of the correlation matrix was performed using the Euclidean distance method, which evaluated the degree of similarity among individual patients in the cohort (Fig. 1A). Eventually, we identified three clusters, each displaying a different set of TMM activities. TERT-related telomerase pathway activity was particularly high in cluster 2, whereas cluster 1 showed high activity in the ALT pathway associated with telomere instability, and cluster 3 showed high activity in the ALT pathway associated with chromatin decompaction (Fig. 1B).

To validate the TMM activity of each sample, we classified the data into four categories (ALT, NDTMM, TEL, and TEL + ALT) based on our previous pan-cancer study [3] (Fig. 1C).

We compared TMM differences between sporadic VS and NF2 VS. ALT HR pathway activity was higher in NF2 VS, and ALT chromatin decompaction pathway activity was higher in sporadic VS. The vestibular schwannoma of different molecular subtypes had different TMM types (Fig. 1D). We investigated the cancer hallmark characteristics of each TMM subtype, and cluster 2 demonstrated more than 50% activity in cancer hallmark pathways, including hedgehog signaling, mitotic spindles, and myogenesis. Cluster 3 showed high activity in notch signaling and WNT beta-catenin signaling, which can be linked to cancer progression via the TMM subtypes (Fig. 1E). For each cluster, we identified significant differentially expressed genes. Cluster 1 was enriched in the TP53 downstream pathway, Cluster 2 in the estrogen signaling pathway, ATM signaling pathway, notch signaling pathway, and TGF beta signaling, and Cluster 3 in neuron projection development. In cluster 2, which showed high activity in the telomerase pathway related to TERT, a higher cancer signaling pathway was confirmed compared with the other clusters. These findings will help predict the prognosis of patients with TMM (Fig. 1F, G, H).

### Tumor immune microenvironment for TMM subtypes

**Fig. 2:**
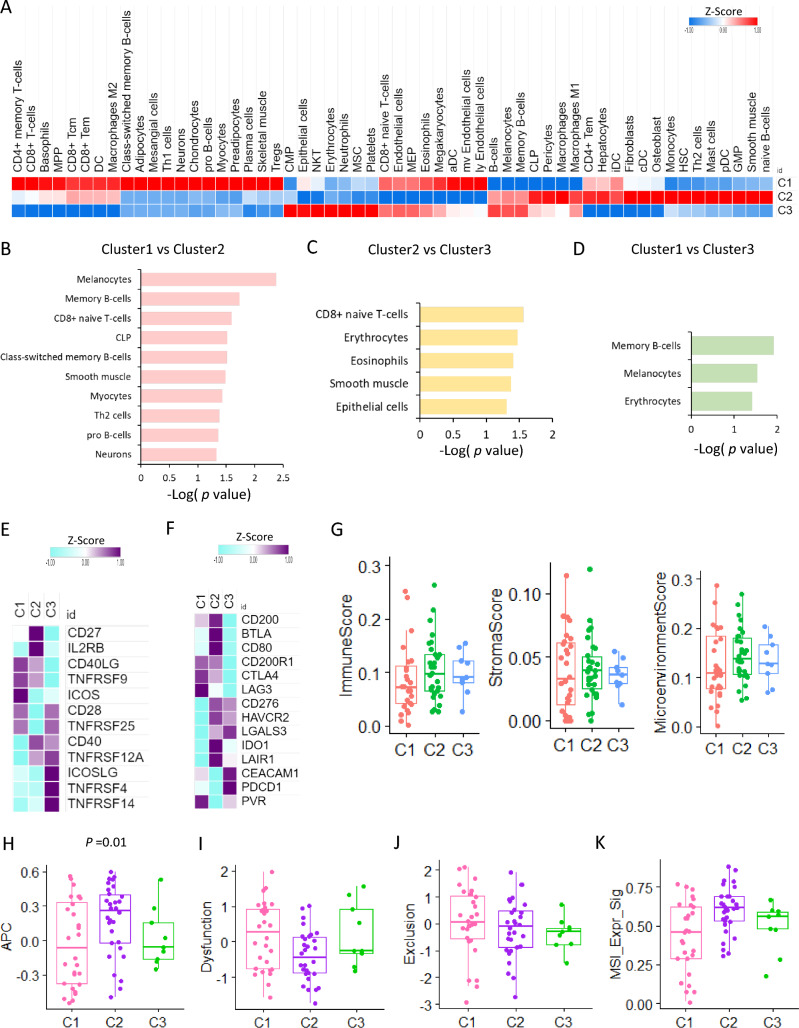
Tumor immune microenvironment for TMM subtypes **A** Heat map of immune cell types enrichment for three clusters. **B** Bar graph of significant difference between cluster 1 and cluster 2 **C** Bar graph of significant difference between cluster 2 and cluster 3 **D** Bar graph of significant difference between cluster 1 and cluster 3 **E** Heat map of immune stimulatory molecules expression for each cluster **F** Heat map of T cell inhibitory molecules expression for each cluster **G** Box plot of immune score, stromal score, microenvironment score for each cluster **H** Box plot of antigen presenting cell (APC) activity for each cluster **I** Box plot of T cell dysfunction for each cluster **J** Box plot of T cell exclusion for each cluster **K** Microsatellite instability (MSI) expression signature for each cluster

We conducted a deconvolution analysis to investigate the enrichment of various cell types in the tumor microenvironment based on TMM subtypes. Telomerase activity was high in cluster 2, but fibroblasts, smooth muscle cells, and naïve B cells were enriched, while ALT-like clusters 1 and 3 showed different patterns. Cluster 1 was enriched in CD8 + T cells, macrophages M2, neurons, and skeletal muscle. Memory B cells, naïve CD8 + T cells, class-switched memory B cells, and pro-B cells were among the cell types that differed significantly between Clusters 1 and 2. Between Cluster 2 and Cluster 3, CD naïve T cells and epithelial cells were enriched in Cluster 3, while memory B cells were enriched in Cluster 3 compared to Cluster 1 (Fig. 2A, B, C, D).

We compared the expression differences of T cell stimulatory and inhibitory molecules by cluster and found that CD27, IL2RB, and CD40 expression were high in cluster 2, while T cell inhibitory molecules were enriched in cluster 2, including CD200, BTLA, CD80, IDO1, and LAIR1 (Fig. 2E, F). For each cluster, the immune, stromal, and microenvironment scores were highest in cluster 2 (Fig. 2G). We also analyzed the immune activity of each cluster as a gene signature related to antigen presenting cells, which was significantly highly activated in cluster 2. T cell dysfunction was lowest in cluster 2, and exclusion did not show significant differences between the clusters. In contrast, the MSI expression signature was highly upregulated in cluster 2 (Fig. 2H, I, J, K).

These findings suggest different tumor immune environments in TMM subtypes, highlighting the need for personalized treatment of patients with vestibular schwannomas (VS) from a precision medicine standpoint.

### Different cell types show distinct TMM activity at the single cell level

**Fig. 3:**
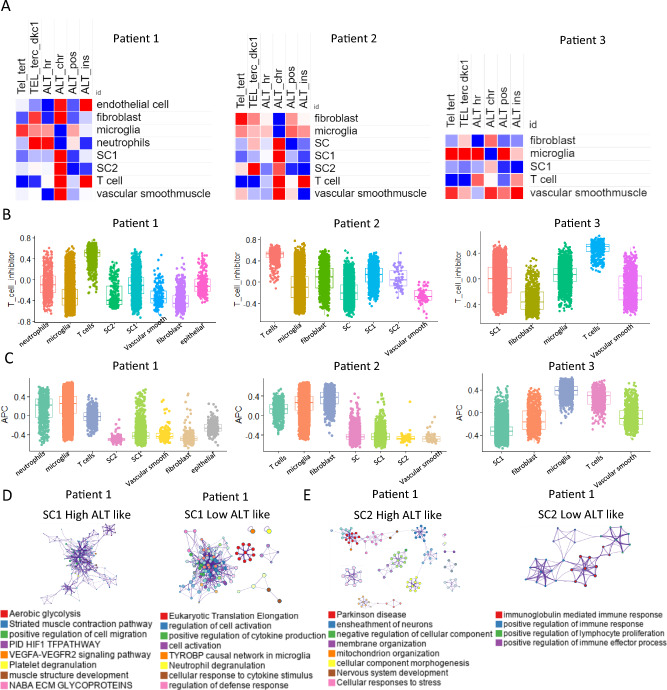

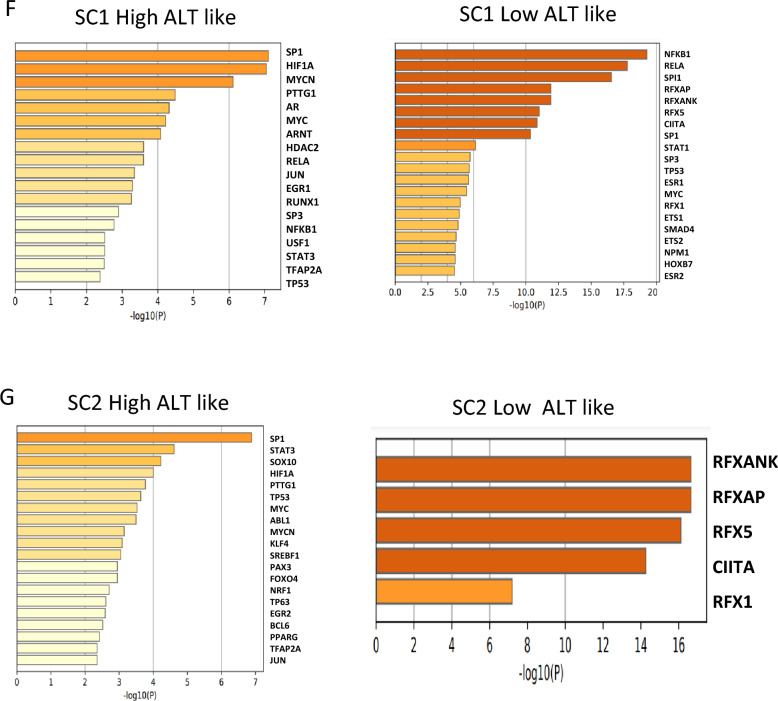
Different cell types show distinct TMM activity at the single cell level **A** Heat map of telomere maintenance mechanism activity for each patient. **B** Box plot for activity of T cell inhibitory molecules for diverse cell types in each patient. **C** Box plot for antigen presenting cell (APC) activity for diverse cell types in each patient **D** Network for enriched biological pathway between high ALT like Schwann cells 1 and low ALT like Schwann cells 1 in patient1. **E** Network for enriched biological pathway between high ALT like Schwann cells 2 and low ALT like Schwann cell 2 in patient1. **F** Bar graph of predicted transcription factor between high ALT like Schwann cells1 and low ALT like Schwann cells1 **G** Bar graph of predicted transcription factor between high ALT like Schwann cells2 and low ALT like Schwann cells2

To overcome the limitations of bulk samples, we utilized single-cell data to analyze TMM pathway activity by cell type. Our study revealed that TMM characteristics were similar in all three patients. Telomerase activity was high in fibroblasts, microglia, neutrophils, and vascular smooth muscle cells, while ALT-like tumor cells were observed in Schwann cells 1, Schwann cells 2, and T cells. Surprisingly, T cells showed almost no telomerase activity, indicating a unique characteristic of these cells (Fig. 3A). Additionally, we investigated T cell inhibitory molecule signature activity and found that Schwann cells had high activity in most cases, while microglia showed high activity in patient 3 (Fig. 3B). Interestingly, we observed that at the single-cell level, microglia showed the highest APC activity compared to other cell types. In patient 2, APC was high in fibroblasts, indicating a high probability of antigen presentation with cancer-associated fibroblasts (Fig. 3C).

To identify the characteristics based on the ALT activity of tumor cells, we divided the Schwann cells of patient 1 into high and low ALT-like cells and enriched each group to analyze biological pathways. We found that the SC1 High ALT of patient 1 had enriched pathways, including aerobic glycolysis, the HIF1 TF pathway, the VEGFA-VEGFR1 signaling pathway [9], and NABA ECM glycoproteins [10], while SC1 low ALT had enriched pathways for cytokine production, neutrophil degradation, and cellular responses to cytokine stimuli (Fig. 3D). The SC2 High ALT of patient 1 was enriched in Parkinson's disease, negative regulation of cellular components, and nervous system development, while the SC2 low ALT had enriched pathways for immunoglobulin-mediated immune responses, positive regulation of immune responses, positive regulation of lymphocyte proliferation, and positive regulation of immune effector processes (Fig. 3E). Transcription factors of each group were found to be common in the high ALT like group of SC1 and SC2 (Fig. 3F, G).

These results are consistent with our previous GBM study, which showed that immune activity was higher in samples with low ALT levels than in those with high ALT levels.

Overall, our study highlights the importance of single-cell analysis in overcoming the limitations of bulk samples and provides insights into the TMM pathway activity by cell type. These findings could help in the development of personalized treatment strategies for patients with vestibular schwannomas based on their individual tumor microenvironments.

### Microglia stemness is positively correlated with telomerase activity at the single cell level

**Fig. 4:**
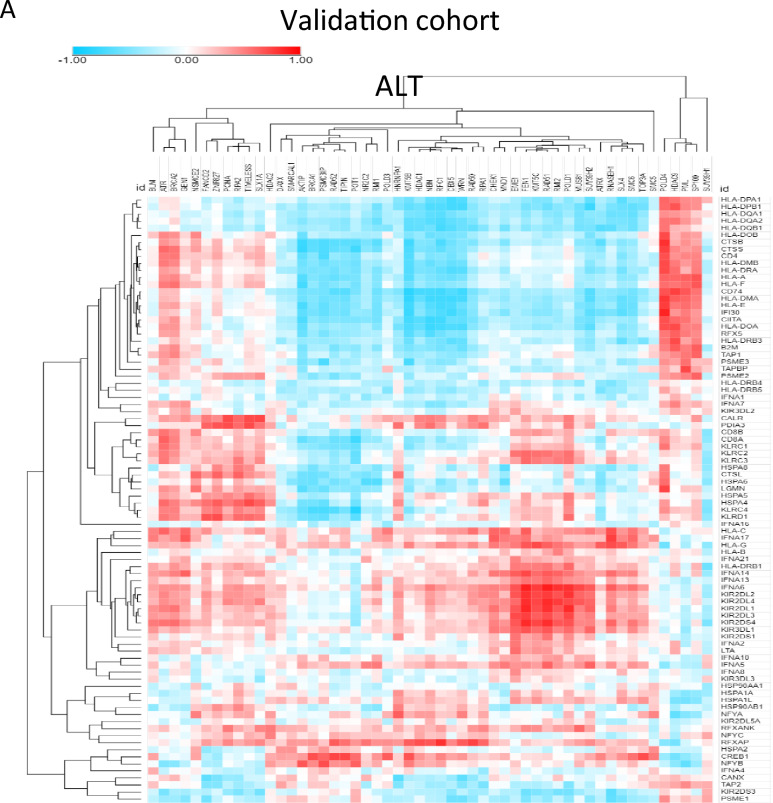

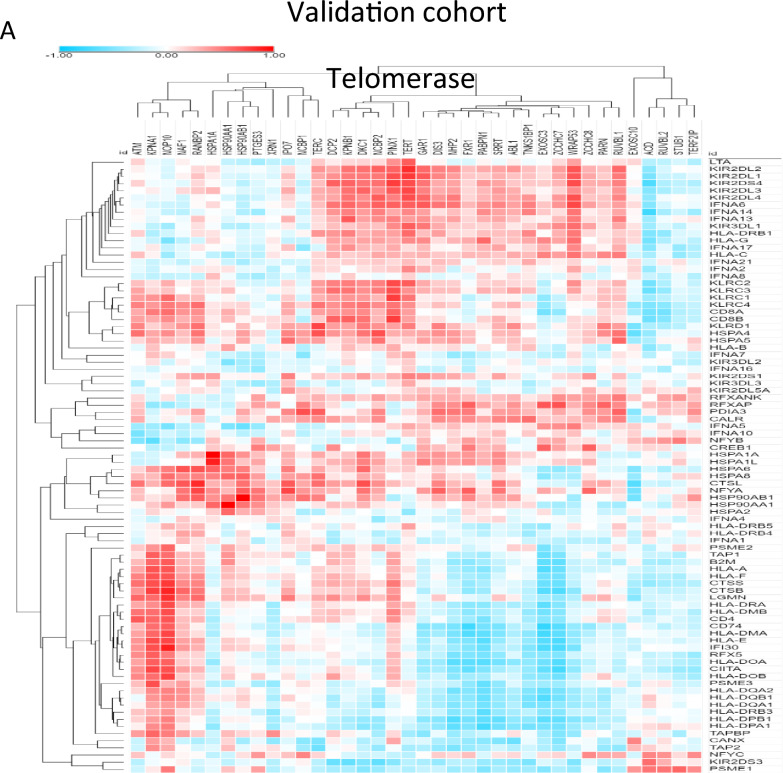

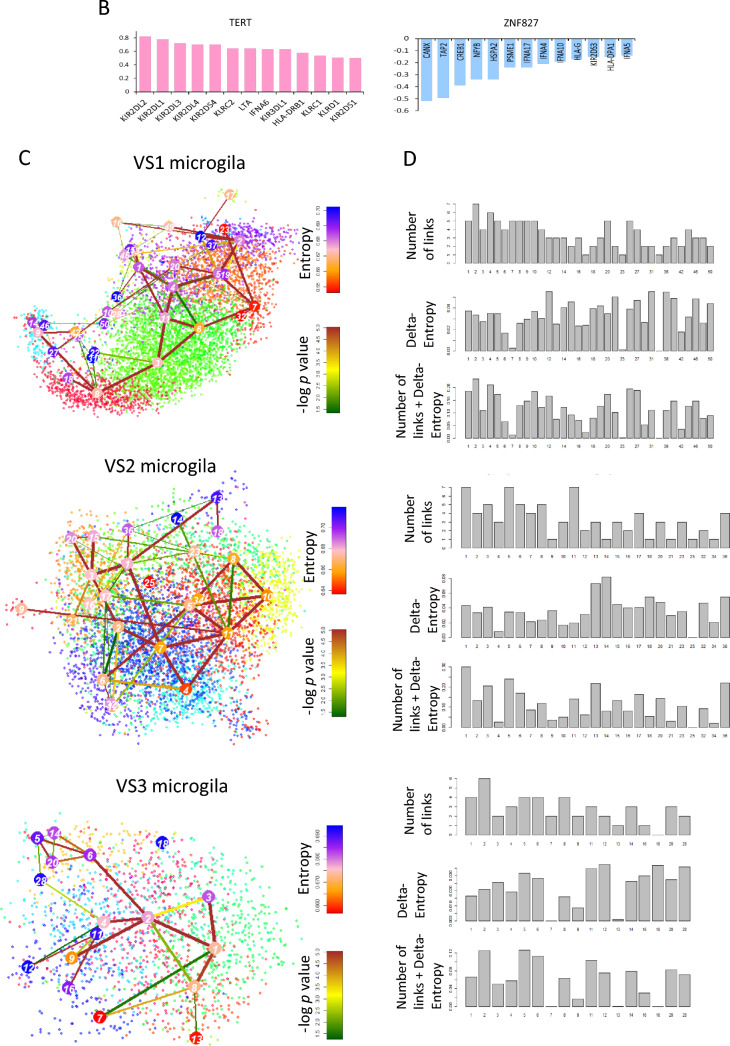

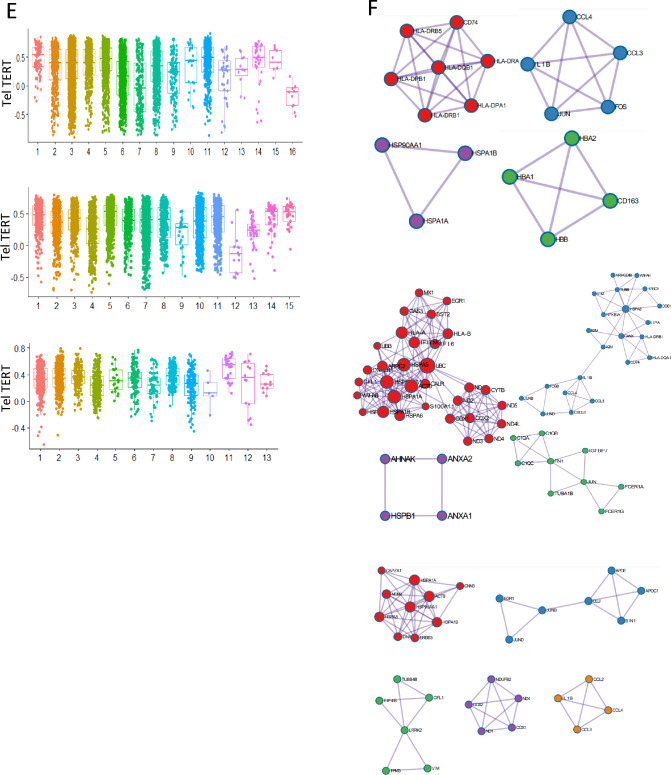
Microglia stemness is positively correlated with telomerase activity at the single cell level **A** Heat map of correlation between ALT/ telomerase and antigen presenting cell (APC) genes **B** Bar graph for correlation between TERT and APC genes and ZNF827 and APC genes **C** t-SNE plot of microglia stemness in each patient [royal blue: high entropy, brown: high (-log *p* value)] **D** Bar graph of stemness for each cluster **E** Box plot of telomerase activity for each stemness cluster (left: patient 1, middle: patient2, right: patient3) **F** Network for protein–protein interaction of enriched in high stemness microglia (left: patient 1, middle: patient2, right: patient3). MCODE efficiently identifies closely linked sections of a protein interaction network, the majority of which relate to recognized molecular complexes, using only connectivity data. Node size indicates importance

To validate our findings, we used the GSE108524 bulk RNA-seq dataset and found a strong correlation between telomerase, ALT, and antigen-presenting cell (APC) genes (Fig. 4A). Specifically, we observed a high correlation between *TERT* and *KIR2DL2*, *KIR2DL1*, and *KIR2DL3* genes (R > 0.6), whereas the ALT chromatin decompaction pathway gene *ZNF827* was negatively correlated with *CANX*, *TAP2*, and *CREB1* (R > −0.5) in most samples (Fig. 4B). Additionally, we confirmed that microglial cells have higher telomerase activity than other cell types, particularly those with high entropy and stemness. Our findings suggest that high telomerase activity is associated with immune activity, and we identified a gene signature that is highly expressed in high-stemness microglial cells (Fig. 4C, D, E).

We used the MCODE algorithm [11] to analyze protein–protein interactions and found that high-stemness microglial cells from patients with VS1 were enriched in antigen processing and presentation of exogenous peptide antigens via MHC class II, the Toll-like receptor signaling pathway, scavenging of heme from plasma, and nitric oxide transport, which were enriched for HSF1-dependent transactivation. Patients with VS2 showed enrichment in cytokine signaling in the immune system, antigen processing and presentation, signaling by interleukins, and classical antibody-mediated complement activation. Patients with VS3 showed enrichment in the HSP90 chaperone cycle for steroid hormone receptors in the presence of ligands, signaling by Rho GTPases, regulation of neuron projection development, and interleukin-10 signaling (Fig. 4F). Our findings provide insights into the high immune activity observed in the high-telomerase patient group, and suggest the possibility of specific targeting in the center for precision medicine via VS patient classification.

## Materials and methods

### Bulk RNA-seq analysis

To analyze telomere maintenance pathway-related signature genes, we employed a comprehensive approach. We utilized bulk RNA-seq data from four cohorts (Gene Expression Omnibus: GSE 141801, GSE108524, GSE39645, GSE54934) and performed gene enrichment tests with one million iterations using the GSVA R package [12]. To improve accuracy, we also employed deconvolution analysis with the xCell web-based tool [13]. To identify differentially expressed gene ontology, we utilized METASCAPE [14]. We expanded our analysis by utilizing each signature gene from MsigDB and employed the TIDE algorithm, a web-based tool for T cell dysfunction and exclusion [15]. To ensure statistical significance, we applied an FDR threshold of < 0.001 and employed multiple testing correction using the Student’s *t*-test. This comprehensive approach allowed us to accurately analyze the telomere maintenance pathway-related signature genes and identify key biological pathways involved in tumorigenesis. However, future studies with larger cohorts may further validate our findings.

### Single cell RNA-seq analysis

To perform single-cell analysis, we utilized the Seurat R package, which is a powerful tool for exploring and analyzing single-cell RNA-seq data. To obtain the single-cell data, we received the data files from three vestibular schwannoma patients from previous studies through email, which were kindly shared by their respective authors [16]. Ethics statement is not available.

We used previously published marker genes to classify the different cell types in the data. For gene enrichment analysis at the single-cell level, we utilized the GSVA R package [12]. Additionally, we used the stemID tool to calculate stemness at the single-cell level, which is a useful metric for identifying stem-like cells in the data [17].

## Discussion

The perpetual existence of cancer cells is contingent upon the preservation of telomere length, which enables unrestricted replication and serves as a safeguard against cellular senescence [18].

In the discipline of cancer research, there are currently two established processes for maintaining telomeres, which are regions of repetitive DNA sequences at the ends of chromosomes. These mechanisms are telomerase activation, observed in around 85–90% of cancer cases, [19] and an alternative lengthening of telomeres (ALT) process that relies on homologous recombination. [20]

Tumor cells have the ability to circumvent the natural process of telomere shortening that occurs with each cell division by utilizing TMMs. In the majority of tumor cells, the reactivation of telomerase is responsible for preserving telomere length (TL). However, a subset of tumor cells achieve immortality through a telomerase-independent mechanism known as alternative lengthening of telomeres (ALT).

In previous pan-cancer studies, we have investigated ALT activity and classified patients into four TMM types. Our experience with this classification system has helped us classify patients with vestibular schwannoma based on their TMM status. In a separate study focusing on GBM, we observed that patients with high ALT activity had a better prognosis than those with low ALT activity [21]. Additionally, we found that the low ALT activity group had higher expression of APC-related genes. When it came to ALT chromatin decompaction, LUAD showed a big difference. Unexpectedly, a better outcome was linked to high ALT chromatin decompaction (p = 0.043). High ALT (p = 0.029) and high telomerase (p = 0.022) also helped GBM patients live longer. Many studies in the past have shown that GBM patients with active telomerase have a worse outlook. These findings are consistent with the TMM tendencies observed in GBM [21]. In particular, an increase in the expression of antigen-presenting cell (APC) signature genes in glioblastoma patients with low amounts of ALT could lead to poor results for the patients. We also confirmed (p = 0.0024) that the risk is higher in the Long TL samples than in the Short TL samples. This shows that the TL lengthening group is more clinically important. [2]

However, in this study, we did not apply the four TMM subtype classification system used in our previous pan-cancer studies [3]. We employed hierarchical clustering to acknowledge the diversity of TMM pathway activity in our study, utilizing unsupervised learning. Our patient cohort was categorized into two groups, those with high telomerase activity and those with high ALT activity. Although the mechanism underlying the difference in activity between the telomerase and ALT groups was unknown in the bulk sample, our single-cell analysis helped to answer this question. Our findings confirmed that among various cell types, microglia are the predominant users of telomerase, with greater APC activity seen in cells with greater stemness. These characteristics were also observed in Schwann cells at the single-cell level. While Schwann cells in the high ALT group displayed cancer hallmark characteristics, those in the low ALT group had antigen-presenting cells such as activated microglia cells. The relevant genes were enriched, and these characteristics were validated in a separate cohort of patients with vestibular schwannomas. In this study, we identified stem-like microglial signature genes through highly activated high-stemness microglial cells, mainly related to APC. These findings can provide valuable insights for implementing precision medicine and personalized patient care [22]. One limitation of our study is the small sample size of patients, which could have resulted in a limited ability to categorize a greater variety of patient groups. Currently, the identification of a direct mechanism underlying tumor cell growth and immune antigen presentation remains challenging. The present study is constrained by the observed connection, necessitating more investigations in subsequent studies to elucidate the underlying mechanism.

## Data Availability

We did not generate new data and all downloaded and used public data. We downloaded bulk RNA-seq from GEO(Gene Expression Omnibus: GSE 141801, GSE108524, GSE39645, GSE54934).
